# Professional nurses’ perceptions and experiences with the implementation of an integrated chronic care model at primary healthcare clinics in South Africa

**DOI:** 10.4102/curationis.v40i1.1708

**Published:** 2017-05-23

**Authors:** Ozayr H. Mahomed, Shaidah Asmall

**Affiliations:** 1 School of Nursing and Public Health, University of KwaZulu-Natal, South Africa; 2 National Department of Health, Civitas Building, South Africa

## Abstract

**Background:**

An integrated chronic disease management model has been implemented across primary healthcare clinics in order to transform the delivery of services for patients with chronic diseases. The sustainability and rapid scale-up of the model is dependent on positive staff perceptions and experiences.

**Objectives:**

The aim of the study was to determine the perceptions and experiences of professional nurses with the integrated chronic care model that has been implemented.

**Method:**

A cross-sectional descriptive survey utilising a self-administered questionnaire was conducted amongst all professional nurses who were involved in delivering primary healthcare services at the 42 implementing facilities in September 2014. Each facility has between four and eight professional nurses providing a service daily at the facilities

**Results:**

A total of 264 professional nurses participated in the survey. Prior to the implementation, 34% (91) of the staff perceived the model to be an added programme, whilst 36% (96) of the staff experienced an increased workload. Staff noted an improved process of care, better level of interaction with patients, improved level of knowledge and better teamwork coupled with an improved level of satisfaction with the work environment at the clinic after implementation of the integrated chronic disease model.

**Conclusion:**

Professional nurses have a positive experience with the implementation of the integrated chronic disease management model.

## Introduction

Primary healthcare (PHC) delivered through PHC clinics was adopted as the modus operandi for healthcare delivery in South Africa (National Department of Health 1997). PHC clinics provide a nurse-led service and are required to provide comprehensive integrated PHC services using a one-stop approach for at least eight hours a day, five days a week (National Department of Health 2000). The services to be offered included Women’s and Reproductive Health; Integrated Management of Childhood Illnesses; Management of Acute Illnesses such as Diarrhoea, Pneumonia, Sexually Transmitted Infections; Adolescent and Youth Health; Expanded Programme for Immunisation; Oral Health; Mental Health; Tuberculosis, Gender Violence; Substance Abuse; Chronic Diseases and Geriatrics; and Rehabilitation Services (National Department of Health 2000).

In order to promote access to the services for the previously marginalised communities, the government embarked on a massive infrastructure development programme increasing the number of available clinics by 1600 to 4200 since 1994 (Harrison 2009), providing free healthcare services initially for pregnant women and children, followed by free services for all patients attending PHC clinics (Harrison 2009). This changed the landscape of PHC services dramatically as the services offered at the PHC level increased.

Despite the increase in services that were offered at PHC clinics, the staff establishments at most facilities have remained the same (Heunis, Van Rensburg & Claassens 2006). HIV and its associated complications further increased the patient load at PHC clinics. Initially, patients were provided with counselling, testing and prophylactic treatment for opportunistic infections at the clinics. However, as the antiretroviral treatment (ART) expanded and the need to initiate new patients on ART grew exponentially, a presidential mandate was issued in 2010 that ART must be made available at all the country’s health facilities and that nurses be trained to prescribe and manage patients on the life-prolonging drugs (Office of the President 2009). The nurse-initiated ART service was introduced as a vertical programme at many facilities and was funded by external agencies. This had the benefit of increasing the number of additional cadres of employees such as counsellors, dieticians, pharmacy assistants, data capturers and medical officers who circulated through clinics. However, these services were exclusively available to HIV-positive patients.

People with HIV are living longer and ageing and are developing non-HIV-related chronic conditions similar to the rest of the population. Some non-communicable diseases are related to the HIV infection itself or to the side effects of some of the medicines used to treat HIV infection (Levitt et al. 2011). This compounded with an ageing population and an ever-increasing burden of non-communicable diseases has the potential to increase the nurse workload at PHC clinics. Therefore, the continued operation of a vertical HIV programme might be unsustainable.

The successful management of chronic diseases requires coordination of services for individuals over an extended time period (Rabkina & El-Sadra 2011).

## Background

South Africa’s National Department of Health has placed a renewed focus on strengthening the management of chronic diseases to increase life expectancy and strengthen the health system’s effectiveness (National Department of Health 2010a). The proposed strategies to target chronic diseases include reorganising and improving the functioning of clinical services with the extension of care for both communicable and non-communicable conditions into communities. This is being implemented through an integrated chronic disease management (ICDM) framework using the re-engineered PHC framework (National Department of Health 2010b).

**FIGURE 1:**
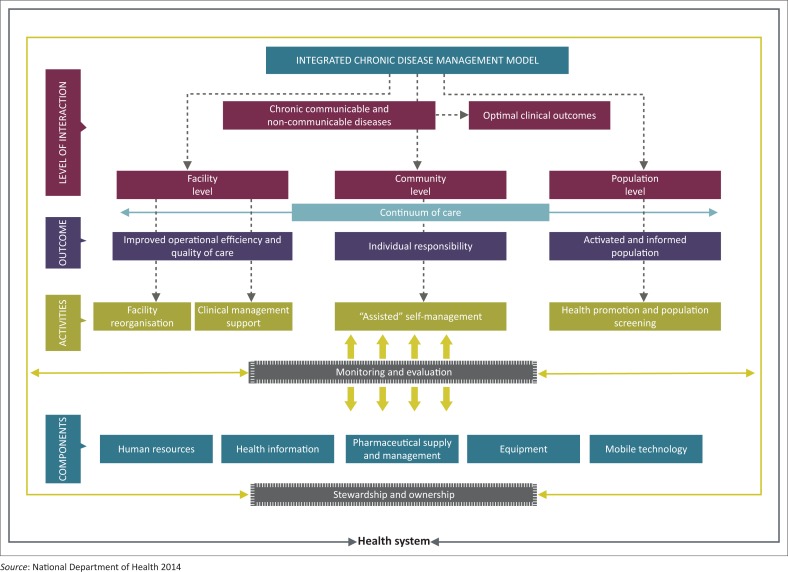
Integrated chronic disease management model for South Africa.

Leveraging the innovations of the HIV programme and utilising the PHC re-engineering framework, the ICDM model was initiated between April 2011 and March 2013 and subsequently scaled up as a vehicle to improve the management of chronic diseases (Asmall & Mahomed 2013) (Figure 1).

The ICDM represents a fundamental shift in the management of patients with long-term chronic diseases evolving from a ‘disease-centred model’ towards a ‘patient-centred care’ approach (International Alliance of Patients’ Organizations 2007), where patients are informed and educated to participate in their own care.

The ICDM consists of four inter-related intervention phases, namely facility reorganisation, clinical supportive management, assisted self-supportive management and strengthening of support systems and structures outside the facility that interact at the health service level, individual patient level and the community or population level to ensure a seamless transition to assisted self-management within the community (see Figure 1).

The clinical supportive management phase of the ICDM includes training in and implementation of Primary Care 101 (PC101) Clinical Guidelines (National Department of Health 2011/2012) as a standardised approach to clinical management and the application of a structured clinical record for patients with chronic diseases supporting the appropriate management of patients according to the predefined algorithms.

### Problem statement

The facility reorganisation component of the ICDM involves the application of the lean-thinking principles to improve patient process flow through the facilities. Patients with chronic communicable and non-communicable diseases receive services at a single point of care from a designated service area. Patients’ clinical records are integrated into a single clinical document and stored in a single location. Patients’ chronic follow-up visits are distributed uniformly across five days of the week as scheduled appointments (Mahomed, Freeman & Asmall 2014). The main outcome of the facility reorganisation component of the ICDM model was to improve the operational efficiency at the facilities. Professional nurses received training with regard to PC101 via an outreach-based training mechanism in order to increase their capacity to manage patients with common diagnostic conditions. Although the ICDM has noble outcomes of improving the operational efficiency at facilities, the success of the implementation and sustainability of the ICDM depends on active staff engagement and positive staff experiences.

## Aim

The aim of the study was to determine the perceptions, experiences and attitudes of PHC professional nurses with the ICDM model that has been implemented across 42 PHC clinics in the Gauteng, Mpumalanga and North West provinces.

### Literature review

The provision of healthcare services occurs in a complex and rapidly evolving environment as a result of an ageing population with multiple complex morbidities, highly advanced and new therapeutic possibilities and the rising expectations of patients in terms of quality and outcomes of care. Healthcare professionals are key components of ensuring the delivery of healthcare services. It is therefore essential that healthcare professionals work within a positive practice environment – a setting that strives to ensure the health, safety and personal well-being of staff; support quality patient care; and improve the motivation, productivity and performance of individuals and organisations (Rondeau & Francescutti 2005).

A study conducted with data from 10 184 nurses and 232 342 surgical patients in 168 Pennsylvanian hospitals showed that the odds of nurses reporting concerns with patient care quality were between 42% and 69% lower in hospitals with better care environments than in hospitals with poorer ones (Aiken et al. 2008).

The nursing practice environment has been shown to be a significant predictor of job satisfaction (Choi et al. 2011). A cross-sectional survey was conducted in 21 hospitals across the Guangdong province in China amongst 1104 bedside nurses in 89 medical, surgical and intensive care units. These results showed that 37% of the professional nurses experienced high burnout, and 54% were dissatisfied with their jobs. Improving nurses’ work environments from poor to better was associated with a 50% decrease in job dissatisfaction and a 33% decrease in job-related burnout amongst nurses (Liu et al. 2012).

Positive changes in the work environment result in higher employee retention rates, which lead to better teamwork, increased continuity of patient care and ultimately improved patient outcomes (Aiken et al. 2008).

## Research method and design

### Context

The study was conducted at 42 ICDM implementing facilities (10 in Dr Kenneth Kaunda District, North West, 15 in the West Rand Health District, Gauteng, and 17 in the Bushbuckridge sub-district within the Ehlanzeni District, Mpumalanga). The facilities have between four and eight professional nurses providing daily services.

### Study design and population

A cross-sectional descriptive study was conducted in September 2014 amongst all professional nurses who were employed for more than six months at the 42 surveyed facilities.

#### Data collection

A self-administered questionnaire was provided to all professional nurses employed at the 42 ICDM initiating clinics. They were requested to complete the questionnaire confidentially in their respective consultation rooms and return the completed questionnaire in a sealed envelope to the operational manager. The questionnaire comprised a single question concerning nurses’ perceptions about the ICDM prior to implementation and six questions on the impact of the ICDM implementation on workload, processes of care, knowledge and scope of practice, teamwork satisfaction levels and interaction with patients. The final question pertained to the professional nurses’ overall impression of and support for the ICDM. All questions were closed-ended requiring a Yes, No or Unsure response.

### Data analysis

Data were processed and analysed using Stata 13.0 (StataCorp 2013). Frequency and descriptive statistics were derived and expressed as numbers and percentages.

### Ethical consideration

The study was conducted within the study assessing the implementation and sustainability of the ICDM for which ethical approval was obtained (BE: 423/13) from the University of KwaZulu-Natal. Permission to undertake the study was obtained from the national, provincial and district departments of health including the facility managers. All data were collected anonymously. Informed consent was obtained from all professional nurses prior to participation.

## Results

### Response rate

Data were received from 91% (38) of the ICDM initiating facilities. A total of 264/280 professional nurses participated in the survey. The majority of the respondents (86%; *n* = 226) were younger than 50 years of age with 58% (*n* = 154) of the respondents having worked fewer than five years at their current facilities (see Table 1).

**TABLE 1:** Frequency distribution of age and service profile of respondents across all facilities (*n* = 264).

Frequency distribution	Number	%
Length of service		
< 5 years	154	58
6–10 years	87	33
> 10 years	23	9
Age category		
< 35 years	103	39
36–50 years	123	47
51–75 years	38	14

### Perceptions regarding the integrated chronic disease management

Prior to the introduction of the ICDM, 61% (*n* = 151) of the professional nurses had a positive perception of the ICDM indicating that the ICDM was not an added programme with no added benefit, whilst 91 (34%) respondents were of the opinion that the ICDM was an added programme with no added benefits. Professional nurses who had been employed for between 6 and 10 years (46%; *n* = 40) and those aged between 36 and 50 years (35%; *n* = 43) had a perception that the ICDM was an added programme with no added benefit (see Table 2).

**TABLE 2:** Frequency table of perceptions, experiences and attitudes towards implementation of the integrated chronic disease management.

Perceptions	< 5 years *n* (%)	6–10 years *n* (%)	> 10 years *n* (%)	Total *n* (%)	< 35 years *n* (%)	36–50 years *n* (%)	51–75 years *n* (%)	Total *n* (%)
I think that the ICDM is an added programme	43 (28%)	40 (46%)	8 (35%)	**-**	36 (35%)	43 (35%)	12 (32%)	**-**
Added to the work load at the facility	49 (32%)	32 (37%)	15 (65%)	96 (36%)	44 (42%)	42 (34%)	10 (26%)	96 (36%)
Improved the process of care at my facility to the benefit of patients	139 (90%)	69 (79%)	15 (65%)	223 (85%)	84 (82%)	102 (83%)	37 (97%)	223 (85%)
Better level of interaction with my patients – getting to know them better	142 (92%)	73 (84%)	15 (65%)	230 (87%)	86 (84%)	108 (88%)	36 (95%)	230 (87%)
The integrated chronic care model has improved my level of knowledge and scope of practice	139 (91%)	74 (85%)	17 (74%)	230 (87%)	83 (81%)	110 (89%)	37 (97%)	230 (87%)
Function as a team in the clinic	139 (90%)	73 (84%)	17 (74%)	229 (87%)	83 (81%)	111 (90%)	35 (92%)	229 (87%)
Increased my level of satisfaction with the work environment	117 (76%)	68 (78%)	12 (52%)	197 (75%)	74 (72%)	91 (74%)	32 (84%)	197 (75%)
Integrated model is great and I fully support its implementation	105 (88%)	73 (84%)	16 (70%)	194 (86%)	84 (82%)	105 (85%)	37 (97%)	226 (86%)

ICDM, integrated chronic disease management.

#### Experience with integrated chronic disease management implementation

Only 36% (*n* = 96) of the respondents experienced an increased workload because of the implementation of the ICDM. The majority of the professional nurses noted an improved process of care (85%; *n* = 223), better level of interaction with patients (87%; *n* = 230), improved level of knowledge (87%; *n* = 230) and better teamwork (87%; *n* = 230) at the clinic with the implementation of the ICDM. Most (75%; *n* = 197) of the respondents experienced an improved level of satisfaction with the work environment after the introduction of ICDM. Professional nurses older than 50 years of age had more positive experiences with the implementation of the ICDM with 97% (*n* = 37) of the respondents noting an improved process of care, 95% (*n* = 36) indicating a better level of interaction with patients, 92% (*n* = 35) indicating an improved level of knowledge and better teamwork at the clinic since the implementation of the ICDM. In all, 84% (*n* = 32) of the professional nurses older than 50 years of age experienced an improved level of satisfaction with the work environment after the introduction of the ICDM.

Staff employed for more than 10 years were less impressed with the ICDM with 65% (*n* = 15) experiencing an increased workload and only 52% (*n* = 12) reporting an increased level of satisfaction with the work environment post-ICDM implementation. However, the majority of the professional nurses with fewer than five years of service noted an improved process of care (90%; *n* = 139), better level of interaction with patients (92%; *n* = 142), improved level of knowledge (91%; *n* = 139) and better teamwork (90%; *n* = 131) at the clinic with the implementation of the ICDM. Most (76%; *n* = 117) of the respondents experienced an improved level of satisfaction with the work environment after the introduction of ICDM.

#### Attitudes towards the integrated chronic disease management

Of the participating professional nurses, 86% (*n* = 226) had a positive attitude towards the ICDM. As many as 88% (*n* = 105) of the respondents with fewer than five years’ service and 84% (*n* = 73) of those with 6–10 years’ service had positive attitudes towards the implementation of the ICDM. In all, 85% (105/123) of the professional nurses between 36 and 50 years and 97 (37/38) of the professional nurses between the ages of 51 and 75 years fully supported the ICDM implementation, whilst 70% (16/23) of the professional nurses with service longer than 10 years in duration fully supported the ICDM (Table 2).

## Discussion

Health service organisations are under pressure for continuous improvement in service provision. However, evidence from the literature indicates that there is a failure rate of up to 70% of organisational change (Draft & Noe 2001) and this is a waste of the scarce resources. Employees are central to quality improvement initiatives and the sustainability of these initiatives.

This study is amongst the first that have surveyed the experiences, perceptions and attitudes of professional nurses who are at the forefront of delivering healthcare services to the public in South Africa. The findings of the study indicate that overall the staff had positive perceptions, experiences and attitudes towards the implementation of the ICDM. It has been suggested that what individuals perceive about their work situation influences their attitudes and behaviour during organisational change (Langton & Robbins 2006). Although 34% (*n* = 91) of the professional nurses who participated in the current study perceived that the ICDM would increase their workload, this did not affect their experience and attitudes negatively towards the ICDM.

The findings from the current study are consistent with an exploratory study conducted in the East London Hospital Complex which showed that nurse managers had a positive attitude towards quality improvement programmes. In this exploratory study positive attitudes were expressed towards the ability of the quality improvement programme to improve the image of the institution, promote team building and accountability, and provide guidance on nursing dynamics (Dondashe-Mtise 2011). In the current study, professional nurses expressed their positive attitudes towards the ICDM. This was also manifested through the positive experiences that the staff had had with the ICDM in terms of improvement of the process of providing care at the facility, improved level of interaction with patients, promoton of team work, enhanced level of knowledge of professional nurses and a better work environment.

Staff members’ feelings, beliefs and attitudes are important components of sustaining any change programme (Maher, Gustafson & Evans 2010). For any quality improvement model to be sustainable, quality improvement efforts will require increased effort and energy from the employees, a belief that improvement is relevant to the patient, team and organisation and a willingness to succeed (Dixon-Woods, McNicol & Martin 2012). Despite the health system challenges, it is evident that professional nurses had a sense that the ICDM model is relevant and were willing to exert the added effort to ensure its success as demonstrated from the 86% (*n* = 226) of employees who indicated that they fully supported the implementation of the ICDM model.

The positive perceptions, experiences and attitudes of the professional nurses can be attributed to meticulous development and implementation of the ICDM (Mahomed & Asmall 2015) as previously described. The key attributes of the implementation included senior management and leadership support, defined case for change, employee involvement in the development and implementation of solutions, clear channels of communication, facility-based implementing agents, training of all staff members, facilitative supervision, sharing of lessons and experiences and benchmarking. This ensured that the employees were actively involved in the ICDM model’s implementation and took ownership of the implementation resulting in positive perceptions, experiences and attitudes as demonstrated in this study.

### Study limitations

Although due diligence was exercised to maintain the scientific integrity of the study, the following are some of the limitations of the study: the study was a cross-sectional study and required responses to specific closed-ended questions. The potential reasons for certain perceptions and experiences were not explored. Secondly, the potential of social desirability bias in the employees’ responses cannot be excluded.

## Conclusion and recommendation

This study indicates that the ICDM has improved the professional nurses’ work environment and as a result has a positive impact on their experiences and attitudes towards the implementation of the ICDM notwithstanding the health system challenges prevalent at PHC clinics in South Africa. It is recommended that prior to, during and after implementation of any quality improvement initiative, change management is integrated within the implementation process to promote a positive staff experience and sustainability of new initiatives.
